# Bilateral subclavius posticus innervated by the lateral pectoral nerve: a cadaveric and ultrasound case report

**DOI:** 10.1007/s00276-026-03829-7

**Published:** 2026-02-13

**Authors:** Athinan Prommahom, Chayanont Jankham, Prin Duangsuwan, Athikhun Suwannakhan

**Affiliations:** 1 Chakri Naruebodindra Medical Institute, Faculty of Medicine Ramathibodi Hospital, Mahidol University, Samut Prakarn, Thailand; 2 Department of Anatomy, Faculty of Science, Mahidol University, Bangkok, Thailand; 3 Human Anatomy Unit, Department of Biomedical Sciences, College of Medicine and Health, University of Birmingham, Birmingham, UK

**Keywords:** Clavicle, Subclavius posticus, Lateral pectoral nerve, Anatomical variation, Ultrasound

## Abstract

**Purpose:**

The subclavius posticus is a well-known variation of the subclavius muscle. This study aims to describe the morphology, innervation, and anatomical relationships of a bilateral subclavius posticus identified in a cadaver, to demonstrate its detection by high-resolution ultrasonography, and to discuss its anatomical and clinical implications.

**Methods:**

Routine dissection of a soft-embalmed adult cadaver, a 54-year-old donor who died from rupture of a cerebral aneurysm, was performed. The infraclavicular and thoracic outlet regions were examined bilaterally. Morphometric data, muscle attachments, and neurovascular relationships were recorded. High-resolution ultrasonography was used to evaluate the contralateral side prior to dissection. Innervation and venous drainage patterns were identified through careful tracing of peripheral nerves and associated vessels.

**Results:**

An accessory muscle consistent with the subclavius posticus was identified bilaterally. On both sides, the muscle originated near the junction of the first rib and costal cartilage and inserted onto the medial aspect of the coracoid process. Ultrasound successfully detected the left-sided muscle prior to dissection. Notably, both muscles were innervated by the lateral pectoral nerve, a finding not previously reported in the literature. Venous drainage was observed through a tributary to the cephalic vein.

**Conclusion:**

This report documents the first bilateral subclavius posticus innervated by the lateral pectoral nerve. These findings broaden the recognized spectrum of subclavius-related variants and highlight the utility of ultrasound for identifying accessory musculature in the thoracic outlet region.

**Supplementary Information:**

The online version contains supplementary material available at 10.1007/s00276-026-03829-7.

## Introduction

The subclavius is a small, cylindrical to fusiform muscle within the clavipectoral triangle, extending from the first rib and its costal cartilage to the inferior surface of the clavicle, where it stabilizes the clavicle and contributes modestly to scapular and clavicular biomechanics [12]. In contrast, the subclavius posticus (SP) represents a supernumerary or accessory muscle slip situated posterior to the non-variant subclavius. The SP most frequently originates from the sternal end of the first rib and runs laterally beneath the clavicle to insert on the scapula [8]. More recently, the term subclavius anticus has been introduced to denote an accessory subclavius variant situated anterior to the non-variant subclavius and attaching to the coracoid process [6]. Clinically, the presence of such variation is important because its location places it directly over the major neurovascular structures traversing the thoracic outlet. As it crosses the superior thoracic aperture, the SP may narrow the costoclavicular interval, directly contacting the brachial plexus, subclavian artery, or subclavian vein [8, 16].

Modern investigations have greatly clarified the morphology and prevalence of the SP. The most comprehensive analysis to date, a meta-analysis of 47 studies, reported an overall prevalence of 4.9%, with rates of 5.1% in cadaveric cohorts and 5.0% in MRI studies [3]. Its distal attachment is highly variable: approximately 71% insert on the superior border of the scapula, ~ 25% on the coracoid process, and ~ 0.9% on the clavicle [3]. In most individuals, the SP is unilateral and asymptomatic, identified incidentally during dissection or imaging [3]. Bilateral occurrence is less common, with only a limited number of cases described. These include bilateral SP reported by Brown [4] and Martin et al. [10] as well as bilateral SPs identified radiologically in two athletes with thoracic outlet syndrome symptoms [5]. Earlier literature also documents bilateral SP in trisomic cadavers, as described by Dunlap et al. [7], Pettersen et al. [11], Ramírez and Barash [13] and Ramirez-Castro et al. [14]. The SP is most commonly innervated by the subclavian nerve (57.6%), followed by the suprascapular nerve (25.8%), with less frequent innervation from the nerve to omohyoid (4.5%), and the accessory phrenic nerve (4.5%) [3]. To our knowledge, innervation of the SP from the lateral pectoral nerve has never been reported.

Therefore, the aim of this study is to document and characterize this rare bilateral SP variant with lateral pectoral nerve innervation, to demonstrate its identification using high-resolution ultrasound, and to discuss its anatomical and clinical implications.

## Case report

**Fig. 1:**
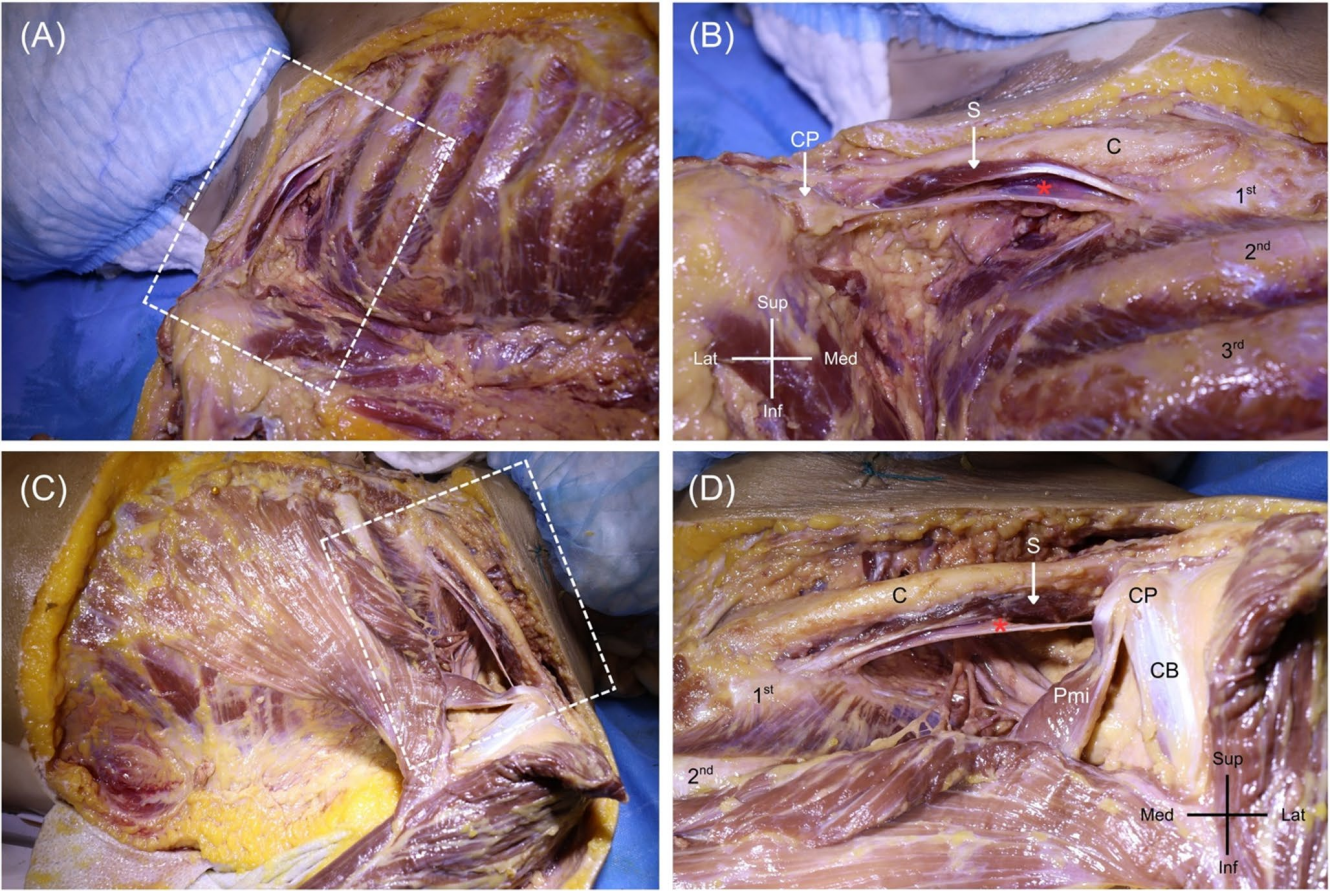
Dissection of the right (**A**, **B**) and left (**C**, **D**) thoracic wall demonstrating the bilateral subclavius posticus (red asterisk) and their respective origins and insertions. C, clavicle; S, subclavius; CP, coracoid process; CB, coracobrachialis; Pmi, pectoralis minor; 1st, first rib; 2nd, second rib; 3rd, third rib

**Fig. 2:**
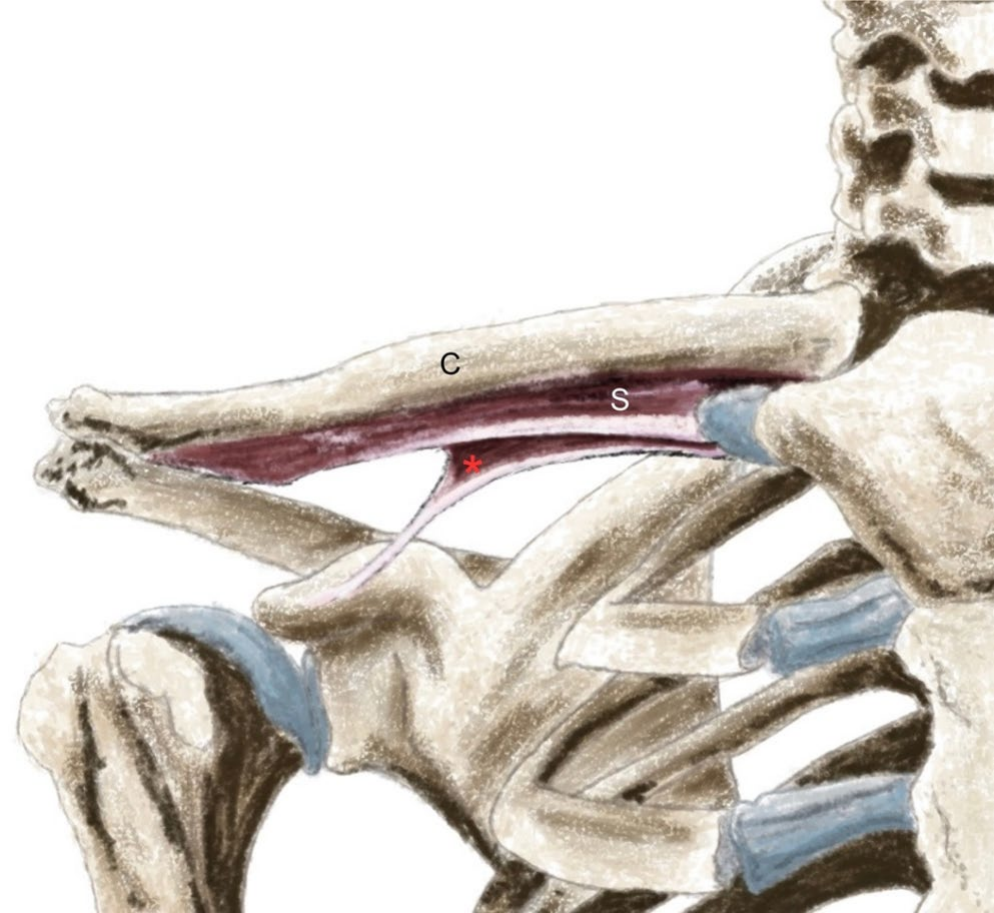
Graphical illustration of the right subclavius posticus (red asterisk) reported in this study. C, clavicle; S, subclavius

The present study was approved by the Human Research Ethics Committee, Faculty of Medicine Ramathibodi Hospital, Mahidol University (certificate of approval number: MURA2025/1006). During routine dissection of a soft-embalmed adult cadaver, a 54-year-old woman who died from rupture of a cerebral aneurysm, at Chakri Naruebodindra Medical Institute, Faculty of Medicine Ramathibodi Hospital, Mahidol University, an anomalous musculotendinous band was noted in the infraclavicular region on both sides. On the right side, after removal of the clavicle mid-shaft and exposure of the first rib and clavicle interface, a distinct triangular muscle belly was identified. This muscle’s proximal attachments were on the first rib (near to the costal cartilage at the sternal end) and the mid-clavicle, crossing dorsolaterally, superficial to the cords/trunks of the brachial plexus, and ultimately inserting into the coracoid process of the scapula (Fig. 1A-B). The muscle’s shape showed that it consisted of muscular tissue at the sternal end and featured a tendinous band at the scapular end. The muscle measured approximately insert measurements (length ~ 93.55 mm, width ~ 9.09 mm, thickness ~ 2.92 mm.) and lay in a plane inferior to the non-variant subclavius muscle. The morphology of this variant (Fig. 1A-B) muscle is consistent with SP. Graphical illustration of the right SP reported in the present study is shown in Fig. 2. Definitive identification of the muscle’s innervation and vascular supply was not possible on the right side.

**Fig. 3:**
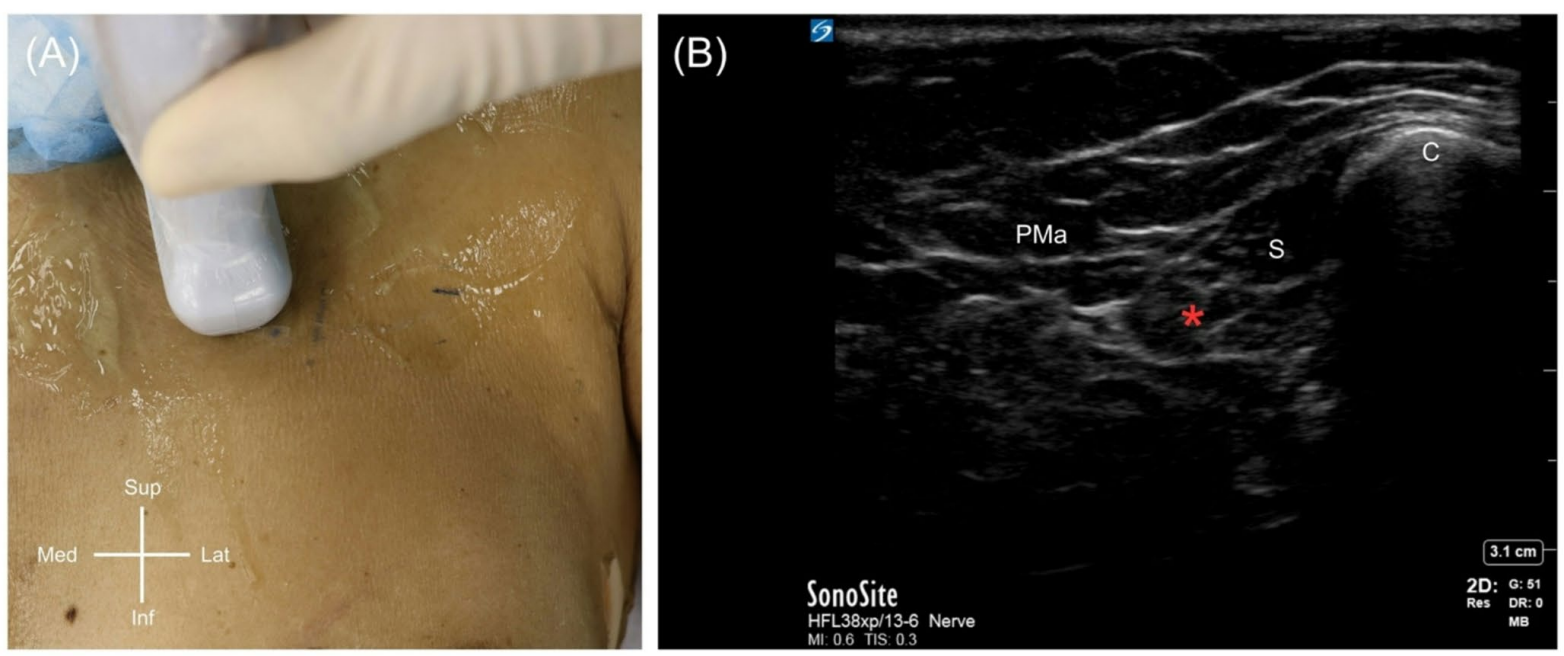
Probe placement for ultrasound examination (**A**) and corresponding high-resolution sonographic visualization (**B**) of the left subclavius posticus (red asterisk). Pma, pectoralis major; C, clavicle; S, subclavius

**Fig. 4:**
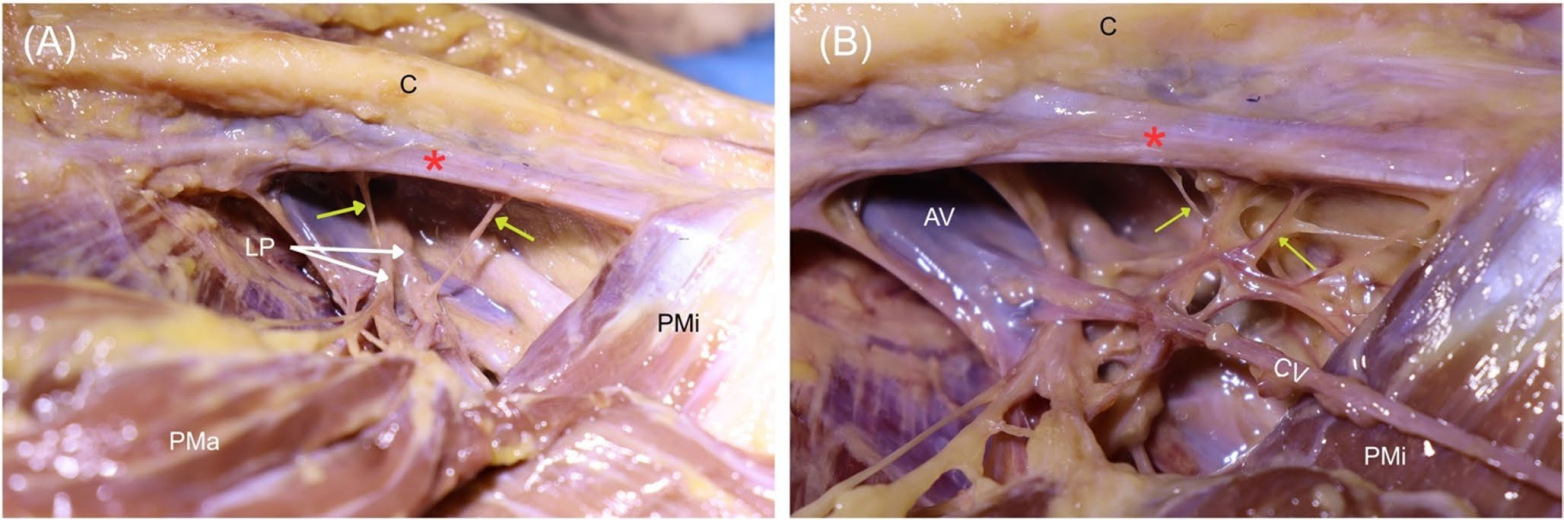
Innervation (**A**) and venous drainage (**B**) of the left subclavius posticus (red asterisk) as identified during dissection. Yellow arrows indicate the nerve branches (left) and venous tributaries (right) of the muscle. C, clavicle; LP, lateral pectoral nerve; Pma, pectoralis major; Pmi, pectoralis minor; AV, axillary vein; CV, cephalic vein

Given the unusual finding, an ultrasound examination (Fig. 3 and Supplementary Video) of the left infraclavicular region of the same donor was performed using high-resolution ultrasonography with a high-frequency linear probe (X-PORTE FUJIFILM Sonosite, WA, USA) with an HFL38 high-frequency linear probe (13–6 MHz). It revealed a hypoechoic muscular structure with a course consistent with SP dissection currently ongoing on the left side. Dissection is presently in progress on the left side, and a preliminary exposure shows that there is a muscle slip that runs from the area where the first rib and clavicle attach (near the junction of the first rib and costal cartilage beneath the typical subclavius muscle) toward the medial aspect of the coracoid process of the scapula. The muscle’s morphology indicated that it comprised muscular tissue at the sternal end and showed a thin tendinous band at the scapular end (Fig. 1C-D). The muscle was measured with the following dimensions: length ~ 98.03 mm, width ~ 11.36 mm, and thickness ~ 4.98 mm. The muscle belly was carefully traced along its course beneath the clavicle to identify its innervation and vascular supply. The innervation was identified as the lateral pectoral nerve, along with a tributary of venous drainage to the cephalic vein (Fig. 4). No gross pathologic features (e.g., vascular narrowing, plexus distortion) were observed on either side of the donor. There was no known history of chromosomal abnormalities of this donor.

## Discussion

### Novelty and terminological issue

The present study reports a bilateral occurrence of SP, identified through dissection and high-resolution ultrasonography. Importantly, this report represents the first documented case in which the SP on both sides is innervated by the lateral pectoral nerve, highlighting its anatomical novelty. A recent scoping review [3] demonstrated that the innervation of the subclavius posticus is variable but predominantly derived from nerves associated with the cervicoscapular region. Across cadaveric series and case reports, the muscle has most frequently been innervated by the subclavian nerve, followed by the suprascapular nerve, with occasional contributions from the nerve to the omohyoid or the accessory phrenic nerve [3]. This pattern is reinforced by pooled meta-analytic data, which show that approximately 57.6% of reported SP receive innervation from the subclavian nerve and 25.8% from the suprascapular nerve, while innervation from the nerve to the omohyoid and accessory phrenic nerve is rare (each ~ 4.5%) [3]. It should be noted that several reports in the literature have described cases in which the innervation could not be identified, largely due to inherent methodological limitations such as tissue preservation. Importantly, all previously documented cases situate the innervation within branches arising from the upper cervical plexus or brachial plexus trunks, reflecting a developmental association with the subclavius–omohyoid or cervicoscapular muscle complex. In contrast, the present study demonstrates bilateral innervation of the SP by the lateral pectoral nerve, a pattern that has not been previously reported. A large-scale cadaveric study is required to determine the true prevalence of SP innervated by the lateral pectoral nerve, as isolated case reports are insufficient to establish its frequency or variability.

The distinction between SP and SA warrants clarification. The term SA was introduced by Diwan et al. [6] to describe a variant of SP attaching to the coracoid process; however, this terminology has appeared only once in the literature. In contrast, SP variants with coracoid process insertion have been reported repeatedly across historical and contemporary studies, as summarized in a recent comprehensive review [3]. These reports indicate that coracoid attachment represents a recognized insertional subtype of SP rather than a distinct anatomical entity. Moreover, in the present study, although the bilateral variant muscles inserted anteriorly onto the coracoid process, the bulk of the muscle belly was located posterior to the clavicle on both gross dissection and ultrasonography, consistent with the classical topography of SP. Accordingly, we interpret our findings within the framework of SP rather than SA.

### Developmental and comparative anatomy

The existence of variations of the subclavius can be understood in light of developmental anatomy. It has been speculated that the SP represents a phylogenetic or atavistic muscle, essentially a remnant of a muscle present in our evolutionary ancestors [15, 16]. Some researchers propose that in certain animals (e.g., some quadrupedal mammals), a muscle exists that connects the first rib or thorax to the scapula, aiding in stabilizing the shoulder girdle [15]. In humans, this might correspond to the SP, which normally is absent or reduced to fibrous tissue. One theory, based on studies in rats, is that the subclavius muscle complex develops from an anlage (primordium) at the junction of the hypobranchial (neck) musculature and the pectoral musculature [3]. During evolution and development, this region undergoes changes (especially as the thorax deepens and the neck differentiates), and the SP may result from an incomplete segregation or a retained muscle slip in this junctional region [16]. Wood [17] suggested that this muscle is well developed in several non-human species, including birds, bats, and moles, and has also been described as particularly prominent in guinea pigs and other rodents. Its function is closely related to an upward extension of the pectoralis minor [17]. The variability in its innervation supports this developmental perspective. In most cases, the SP is innervated by the nerve to subclavius, indicating it shares a common developmental origin with the regular subclavius muscle [3]. However, there are reports of SP being innervated by other nerves, such as the suprascapular nerve, as in Forcada et al.’s surgically verified case [8], or even the nerve to mylohyoid in rare instances [3]. The suprascapular nerve typically supplies supraspinatus and infraspinatus musculature, so its involvement suggests the muscle can integrate into a different nerve territory in select cases. Interestingly, the presence of an accessory phrenic nerve has been noted in conjunction with SP in several cases. This may be because the C5 nerve root contributes to both the phrenic nerve and the nerve to subclavius; a developmental link might cause an anomalous muscle like SP to be associated with an accessory phrenic nerve branch [2, 15]. A case of SP innervated by the omohyoid nerve, a muscular branch from the ansa cervicalis, is a very unusual scenario and has been reported once by [2, 15]. Such anomalies in innervation could be attributed to the shifting and migration of muscle primordia and nerve fibers during embryogenesis. In the present study, we observed that the SP was innervated by the lateral pectoral nerve (Fig. 3A). This finding raises the possibility that this variant muscle may derive from, or at least partially integrate into, the pectoralis major–clavicular head muscle complex, which is normally innervated by the lateral pectoral nerve. However, this developmental relationship remains speculative and warrants further investigation.

### Clinical significance

The SP has significant implications for thoracic outlet syndrome (TOS), particularly neurogenic forms. The muscle commonly passes directly over the brachial plexus, and in several documented living patients has been identified as the principal compressive structure [5, 16]. In the influential case by Smayra et al. [16], MRI demonstrated brachial plexus compression between the SP and scalenus anterior during arm elevation, with complete symptom resolution after muscle excision and first rib resection. The association between SP and TOS has been well documented in the literature; however, recent perspectives have emphasized the importance of precise terminology. In a recent editorial, Abraham et al. [1] highlighted that asymptomatic neurovascular compression should not be equated with thoracic outlet syndrome, and proposed a clearer distinction between *thoracic outlet compression* and *thoracic outlet syndrome*, the latter requiring the presence of clinical symptoms and/or complications. In the present study, although the SP was observed to course in close proximity to the brachial plexus and subclavian vessels, no gross evidence of compression, distortion, or secondary pathological changes was identified, nor was any clinical history available. Therefore, our findings are best interpreted as demonstrating anatomical thoracic outlet compression rather than TOS per se. Our case carries additional significance because of the multimodal identification of the subclavus variations. Ultrasound is increasingly recognized as a powerful, non-invasive tool for the evaluation of thoracic outlet structures, particularly the brachial plexus, pectoralis minor, scalene muscles, and anomalous slips. Detecting the SP ultrasound prior to dissection reiterates its potential value for preoperative mapping, diagnosis of dynamic neurovascular compression, and screening for muscular anomalies that might influence surgical planning in thoracic outlet decompression procedures.

## Conclusion

This study documents a rare bilateral manifestation of the SP, identified through combined dissection and high-resolution ultrasonography, and represents the first reported case in which the muscle was innervated by the lateral pectoral nerve. The unique morphology and innervation pattern expand the known spectrum of subclavius-related variants.

## Supplementary Information

Below is the link to the electronic supplementary material.

Supplementary Material 1

## Data Availability

The data that support the findings of this study are available from the corresponding author, A.S., upon reasonable request .
